# Artemisinin Derivatives Inhibit Non-small Cell Lung Cancer Cells Through Induction of ROS-dependent Apoptosis/Ferroptosis

**DOI:** 10.7150/jca.57054

**Published:** 2021-05-13

**Authors:** Qiuting Zhang, Huimei Yi, Hui Yao, Lu Lu, Guangchun He, Mi Wu, Chanjuan Zheng, Ying Li, Sisi Chen, Lewei Li, Hongyuan Yu, Guifei Li, Xiaojun Tao, Shujun Fu, Xiyun Deng

**Affiliations:** 1 Key Laboratory of Translational Cancer Stem Cell Research, Hunan Normal University, Changsha, Hunan 410013, China.; 2 Departments of Pathology and Pathophysiology, Hunan Normal University School of Medicine, Changsha, Hunan 410013, China.; 3 Key Laboratory of Study and Discovery of Small Targeted Molecules of Hunan Province, School of Medicine, Hunan Normal University, Changsha 410013, China.

**Keywords:** Non-small cell lung cancer, Artemisinin derivatives, Ferroptosis, Apoptosis, Caner therapy.

## Abstract

Non-small cell lung cancer (NSCLC) is one of the major cancer-related causes of morbidity and mortality worldwide. Despite the progress in lung cancer treatment, there is still an urgent need to discover novel therapeutic agents for NSCLC. Natural products represent a rich source of bioactive compounds. Through a natural compound library screening assay, we found that a group of anti-insect drugs had significant inhibitory effect on the proliferation of NSCLC cells. Among the anti-insect drugs, two derivatives of artemisinin, i.e., artesunate (ART) and dihydroartemisinin (DHA), a group of well-known anti-malarial drugs, have been shown to possess selective anti-cancer properties. Mechanistically, we found that ART and DHA induced apoptosis of A549 cells as evidenced by decreased protein level of VDAC and increased caspase 3 cleavage. Furthermore, cystine/glutamate transporter (xCT), a core negative regulator of ferroptosis, was downregulated by ART and DHA. The mRNA level of transferrin receptor (TFRC), a positive regulator of ferroptosis, was upregulated by ART and DHA. ART/DHA-induced apoptosis and ferroptosis in NSCLC cells were partly reversed by N-Acetyl-L-cysteine (NAC), a ROS scavenger, and ferrostatin-1, a ferroptosis inhibitor, respectively. These results suggest that artemisinin derivatives have anti-NSCLC activity through induction of ROS-dependent apoptosis/ferroptosis. Our findings provide the experimental basis for the potential application of artemisinin derivatives as a class of novel therapeutic drugs for NSCLC.

## Introduction

Lung cancer is the most frequently diagnosed cancer worldwide, which accounts for a quarter of all cancer deaths around the world 1. Histopathologically, non-small cell lung cancer (NSCLC) accounts for approximately 85% of lung cancers 2. The main treatment modalities for NSCLC include surgery, radiotherapy, and chemotherapy 3. Despite the progress in lung cancer treatment, there is still an urgent need to discover novel therapeutic agents for NSCLC due to the overall poor prognosis of NSCLC patients.

Natural products have a wide range of clinical efficacy, including cholesterol reduction, anti-malaria and anti-cancer applications. In recent years, more than half of anti-cancer drugs are discovered from natural sources such as plants, fungus, and animals 4. Some examples of anti-cancer natural products of plant origin include vincristine, taxol, and azithromycin that can treat different types of cancer 5, 6. Drug repositioning is the exploration of new clinical indications of approved drugs. As a cost-effective approach to new drug discovery, drug repositioning is receiving more attention compared with the traditional novel therapeutic agent's discovery strategy. Patients benefit from drug repurposing that promote access to successful treatment and low safety risks 7, 8. There are many successful cases of repositioning of existing drugs, such as acetylsalicylic acid, thalidomide, sildenafil and dimethylfumarate. Acetylsalicylic acid (aspirin), the earliest successful example of drug repositioning, was first marketed as an analgesic, but it was repositioned as an anti-platelet aggregation agent at low doses in the 1980's 9. Recently, the anti-tumor effect of artemisinin derivatives in malignant cancers such as lung cancer, colon cancer, nasopharyngeal cancer, and glioma has been widely recognized 10-12. Several studies revealed that artemisinin derivatives induced migration and invasion of cancer cells 13-15. Also, artemisinin derivatives have recently been reported to be a potential direct inhibitor of STAT3 16, 17. Nevertheless, the detailed anti-tumor mechanism of artemisinin derivatives in NSCLC is elusive.

In this paper, we screened a natural product library to identify novel promising agents for NSCLC and found that a group of artemisinin derivatives had potent anti-NSCLC efficacy. Mechanistically, we demonstrated that artemisinin derivatives exerted anti-NSCLC activity through induction of ROS-dependent apoptosis/ferroptosis. This study provides solid experimental basis for the potential application of artemisinin derivatives as a class of novel therapeutic drugs for NSCLC.

## Materials and methods

### Antibodies and reagents

Antibody against cleaved caspase 3 was obtained from Cell Signaling Technology (Danvers, MA, USA). Antibody against xCT was purchased from Sangon Biotech (Shanghai, USA). Antibody against β-actin and tubulin were purchased from Bioworld Technology (Philadelphia, PA, USA). Anti-rabbit IgG (H+L) affinity purified secondary antibody was obtained from Vector Laboratories (Burlingame, CA, USA). Artesunate, dihydroartemisinin, N-Acetyl-L-cysteine and ferrostatin-1were acquired from Selleck (Houston, TX, USA). RPMI-1640 medium was purchased from HyClone (Waltham, MA, USA). Fetal bovine serum (FBS) was purchased from Biological Industries (Cromwell, CT, USA). 2′,7′-Dichlorofluorescin diacetate was purchased from Sigma-Aldrich (St. Louis, MO, USA).

### Cell culture

NCI-H1299, A549, LTEP-a-2, NCI-H23, and NCI-H358 NSCLC cell lines were obtained from the Shanghai Institute of Biochemistry and Cell Biology (Shanghai, China). These cells were cultured in RPMI-1640 medium supplemented with 10% FBS. Cells were incubated in a humidified atmosphere with 5% CO_2_ at 37°C.

### Alamar Blue cell viability assay

To evaluate the inhibition of artemisinin derivatives on NSCLC cells, we performed Alamar Blue cell viability assay kit (G8081, Promega, Madison, WI, USA). The cells seeded in 96-well plates (7 × 10^3^ cells/well) were allowed to adhere overnight. After 24 h, the cells were treated with ART or DHA (1, 3, 10 µM) at 21% O_2_ for 72 h. 20 μL CellTiter Blue reagent was added to each well and the cells incubated for 2-8 h at 37℃. Absorbance at 530/590 nm was measured by using a Synergy 2 Automatic Microplate Reader (BioTek, Winooski, VT, USA).

### Natural compound screening assay

A natural product library, which contained 173 commonly used naturally occurring compounds, was purchased from Selleck (Cat # L1400). We performed the screening assay on NCI-H1299, A549, LTEP-a-2, NCI-H23, and NCI-H358 cell lines according to the manufacturer's recommendations. Briefly, the cells seeded in 96-well plates (1.0 × 10^5^ cells/well) were allowed to adhere overnight. Next day, the cells were treated with natural compounds each at a 5 or 10 µM concentration and grown at 21% O_2_ for 72 h. Cell proliferation was detected by using an Alamar Blue cell viability assay kit as described below.

### Apoptosis analysis

The cells seeded at a density 2 × 10^5^ in 6-cm dishes and allowed to adhere overnight were treated with the drug or vehicle for 72 h. After intervention, the cells were subjected to apoptosis detection using the FITC annexin V/PI apoptosis detection kit (BD Pharmingen, San Jose, CA, USA) according to the manufacturer's protocol. A BD FACS CantoⅡflow cytometer and Flow Jo software were used to analyze cell apoptosis.

### Immunofluorescence assay

Immunofluorescence was performed on A549 and NCI-H1299 cells seeded on coverslips, which were placed in 12-well plates treated with 10 μM ART or DHA for 72 h. After wash once with PBS and fixation, the cells were blocked with 1% goat serum for 20 min at room temperature. Then, the cells were permeabilized with 0.25% Triton-X100 for 5 min. After blocking with blocking solution for 30 min, the cells were incubated with primary antibody (1:50) followed by incubation with Alexa Fluor conjugated secondary antibody (1:100). The images, which were acquired using an identical acquisition time for all samples, were analyzed using a Leica DM3000 microscope.

### Protein harvest and western blot analysis

The cells were seeded in 6-cm dishes (2 × 10^5^ cells/dish) and incubated overnight, followed by treatment with ART or DHA (10 µM) for 72 h. Total proteins were extracted from the cells using RIPA buffer. The protein samples were resuspended in SDS-PAGE loading buffer and heated at 95℃ for 5 min. 25 μg proteins were separated by 12% SDS polyacrylamide gel electrophoresis (SDS-PAGE) and transferred onto PVDF membranes (Millipore, Billerica, MA, USA). After blocking in 5% BSA, the membrane was probed with the respective primary antibody at the recommended dilution overnight at 4℃ followed by secondary antibody incubation and enhanced chemiluminescence (ECL) detection. β-actin on the same membrane was probed and used as a loading control.

### qRT-PCR assay

Total RNAs were isolated from treated cells using Trizol reagent (Invitrogen, Carlsbad, CA, USA). cDNA was generated by iScript^TM^ Reverse Transcription Supermix for qRT-PCR (Bio-Rad). The mRNA levels of TFRC and β-actin were measured by qRT-PCR in 96-well plates with the MiniOption real-time PCR system (Bio-Rad, Shanghai, China) by using 2x NovoStart SYBR qPCR SuperMix Plus (Novoprotein, Shanghai, China). The relative expression levels of mRNA were calculated using the following formulas: ΔΔCt=ΔCt test - ΔCt control, fold change=2-ΔΔCt. The results were from at least three biological replicates. The primers used were as follows: transferrin receptor (TFRC), forward: 5'-TCGTGAGGCTGGATCTCAAAA-3', reverse: 5'-CCTTACTATACGCCACATAACCC-3'; β-actin, forward: 5'-CTGGAACGGTGAAGGTGACA-3', reverse: 5'-AAGGGACTTCCTGTAACAATGCA-3'.

### Reactive oxygen species analysis

Drug treatment and cell collection were performed in the same way as for apoptosis analysis. After collection, the cells were stained with 2′,7′-Dichlorofluorescin diacetate (DCFH-DA) for 45 min in the dark at room temperature. After wash, the cells were resuspended in PBS and fluorescence measured at 480 nm excitation and 535 nm emission by flow cytometry 18.

### Bioinformatics analyses of expression level of TFRC and VDAC and lung cancer patient survival

To explore the gene and protein expression level of molecules (TFRC, VDAC), we analyzed the lung cancer and normal clinical samples from UALCAN database (http://ualcan.path.uab.edu/). Then, we used GEPIA 2 database to analyze the overall survival rate of lung cancer patients with high and low expression of TFRC and VDAC (http://gepia2.cancer-pku.cn/#index).

### Statistical analysis

The quantitative data obtained from at least three independent experiments were presented as mean ± SEM and analyzed by student's* t*-test. *P* < 0.05 was considered statistically significant.

## Results

### Screening for natural products with therapeutic potential for NSCLC

**Figure 1:**
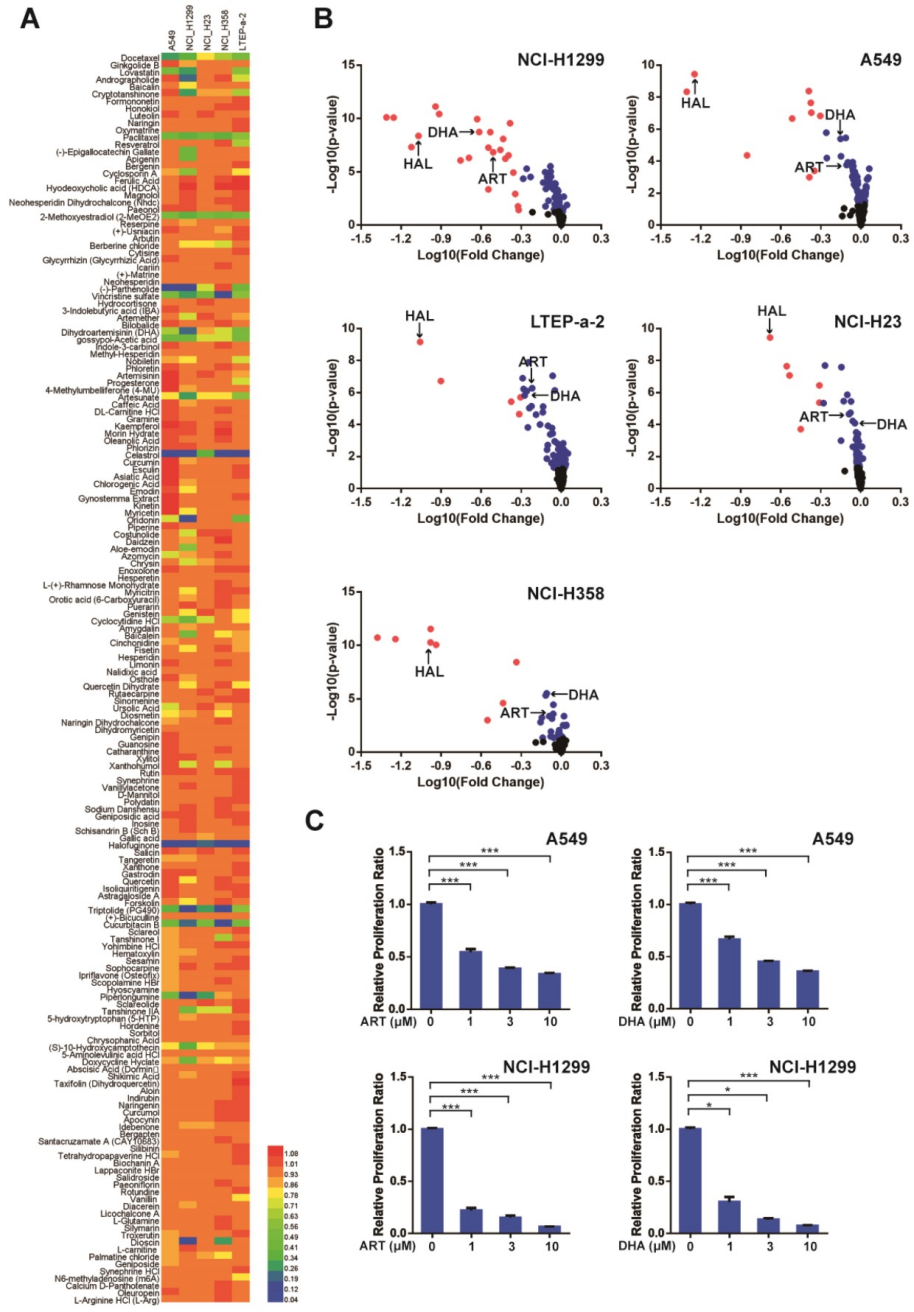
** Artemisinin derivatives significantly inhibit the proliferation of NSCLC cells. (A)** The color of the heatmap represented the relative proliferation ratio of the five NSCLC cell lines treated with each of the 173 natural compounds from the natural product library.** (B)** For screening, the 173 natural compounds were added to NSCLC cells (A549, NCI-H1299, NCI-H23, NCI-H358, and LTEP-a-2) each at 10 μM final concentration for 72 h. DHA: dihydroaremisinin, ART: artesunate, HAL: halofuginone. Red: *P* < 0.05, Fold change < 10^-0.3^; Blue: *P* < 0.05, Fold Change > 10^-0.3^; Black: *P* > 0.05.** (C)** Artemisinin derivatives (ART and DHA) significantly inhibited the proliferation of A549 and NCI-H1299 cells in a concentration-dependent manner. ***P < 0.001, *P < 0.05.

In order to identify natural compounds that inhibit NSCLC cells, we performed a natural compound library screening assay**.** The results showed that about two-thirds of the compounds did not have a strong inhibitory effect on the proliferation of NSCLC cells (**Figure 1A, Figure 1B**). Then, the top 13 natural compounds that had significant inhibitory effect on NSCLC cells were screened out and divided into five categories according to their mechanisms. On the top list was a group of microtubule depolymerization inhibitors which have been used in the clinic for tumor treatment. The second class was a group of anti-insect drugs including ART, DHA, and halofuginone. Among these, ART and DHA are artemisinin derivatives which belong to a class of anti-malarial drugs. We then investigated the inhibitory effect of artemisinin derivatives on NSCLC cells and found that both ART and DHA inhibited NSCLC cells in a concentration-dependent manner **(Figure 1C)**. These results indicated that artemisinin derivatives have the potential to be used as novel therapeutic drugs for NSCLC.

### Artemisinin derivatives induce apoptosis and ferroptosis in NSCLC cells

**Figure 2:**
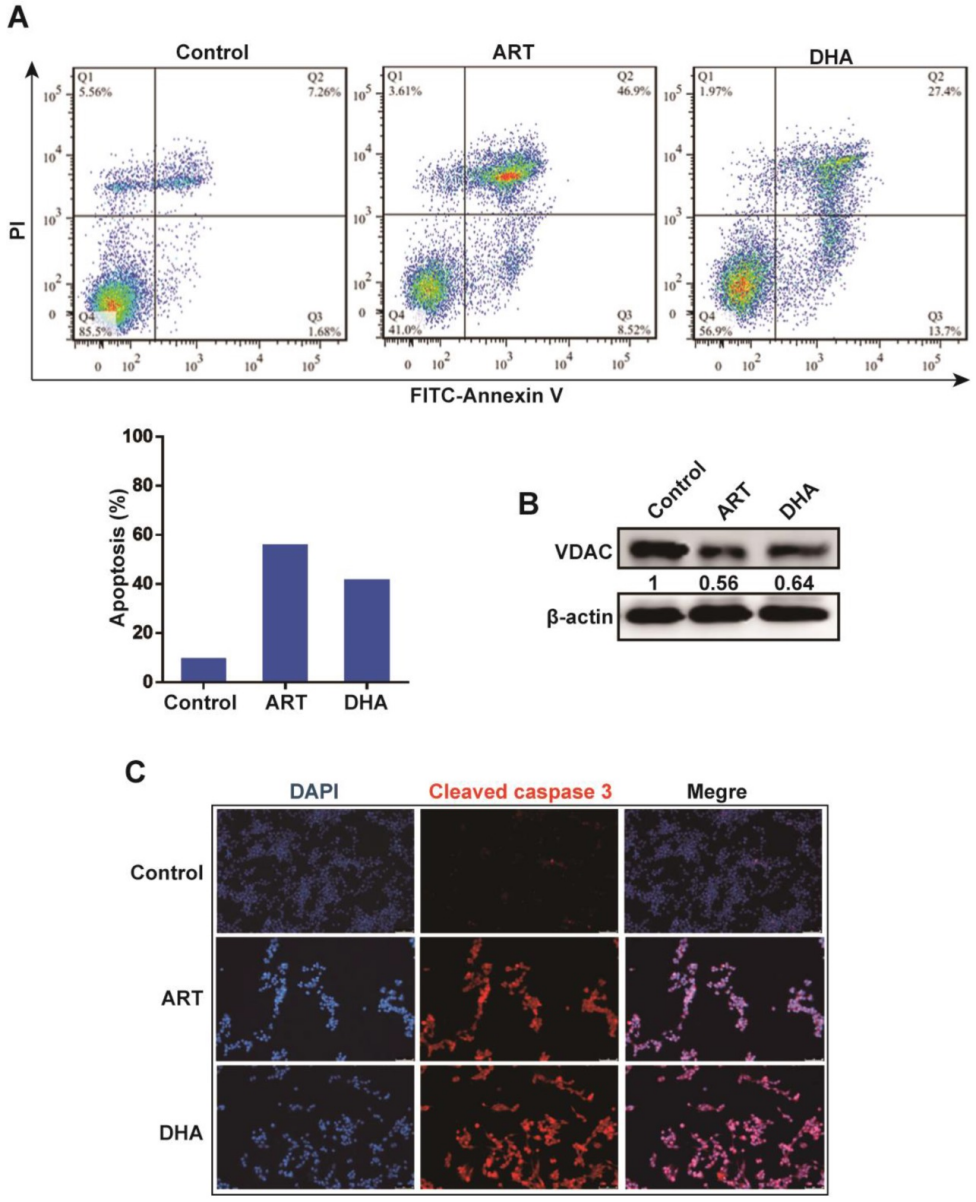
** Artemisinin derivatives induce apoptosis via the mitochondrial-mediated pathway. (A)** ART/DHA significantly induced apoptosis of A549 cells. A549 cells were treated with ART or DHA (10 μM) for 72 h. **(B)** The protein level of VDAC was decreased by ART or DHA normalized to β-actin. A549 cells were treated with ART or DHA (10 μM) for 72 h. **(C)** The protein level of cleaved caspase 3 was downregulated by ART/DHA. A549 cells were treated with ART or DHA (10 μM) for 72 h and immunofluorescence was performed to detect cleaved caspase 3 (red). DAPI was used as a counterstain for the nucleus.

We next investigated the mechanism underlying the inhibitory effect of artemisinin derivatives on NSCLC cells. Apoptosis is an extremely complex, precisely regulated, protease-dependent molecular cascade reaction process. Compared with the control group, both ART and DHA induced apoptosis in A549 cells as revealed by flow cytometry (**Figure 2A**). Western blot analysis showed that ART and DHA respectively downregulated the protein level of voltage-dependent anion channel (VDAC), one of the components of the mitochondrial permeability transition pore (PTP) (**Figure 2B**). Furthermore, immunofluorescence-confocal microscopy revealed that ART and DHA noticeably increased the level of cleaved caspase 3 in A549 cells (**Figure 2C**).

**Figure 3:**
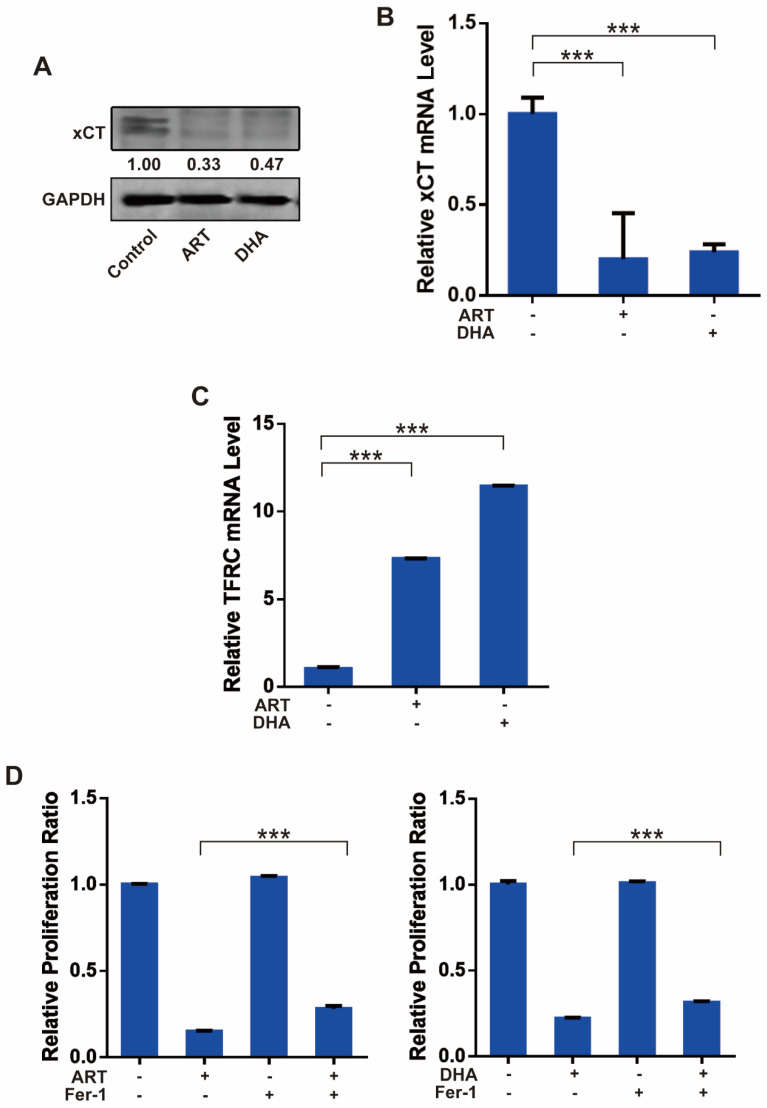
** Artemisinin derivatives inhibit NSCLC cells through induction of ferroptosis. (A)** The protein level of xCT was decreased by ART or DHA normalized to GAPDH. A549 cells were treated with ART or DHA (10 μM) for 72 h.** (B)** The mRNA level of xCT was significantly decreased by ART/DHA. A549 cells were treated with ART or DHA (30 μM) for 48 h.** (C)** The mRNA level of TFCR was significantly increased by ART/DHA. A549 cells were treated with ART or DHA (30 μM) for 48 h.** (D)** The inhibitory effect of ART/DHA on cell proliferation was partly reversed by Fer-1. A549 cells were treated with ART or DHA (10 μM) and Fer-1 (3 μM) for 72 h. ***P < 0.001.

Then, we investigated whether ART/DHA also induces ferroptosis in NSCLC cells. The protein and mRNA level of cystine/glutamate transporter (xCT), which is responsible for the import of cystine and export of glutamate across the cell membrane, were markedly decreased by ART and DHA in A549 cells (**Figure 3A, Figure 3B**). In addition, the mRNA level of TFRC, a cell membrane receptor responsible for the uptake of iron into the cell, was robustly upregulated by ART and DHA (**Figure 3C**). We further used ferroptosis inhibitor ferrostatin 1 (Fer-1) to confirm the involvement of ferroptosis in ART/DHA-induced cell inhibition. We found that the inhibitory effect of ART/DHA on the proliferation of A549 cells was partly reversed by Fer-1 (**Figure 3D**). Our results showed that the inhibitory effect of ART/DHA on NSCLC cells was indeed at least partly through the induction of ferroptosis.

### ROS is a key regulator of ART/DHA-induced apoptosis and ferroptosis

**Figure 4:**
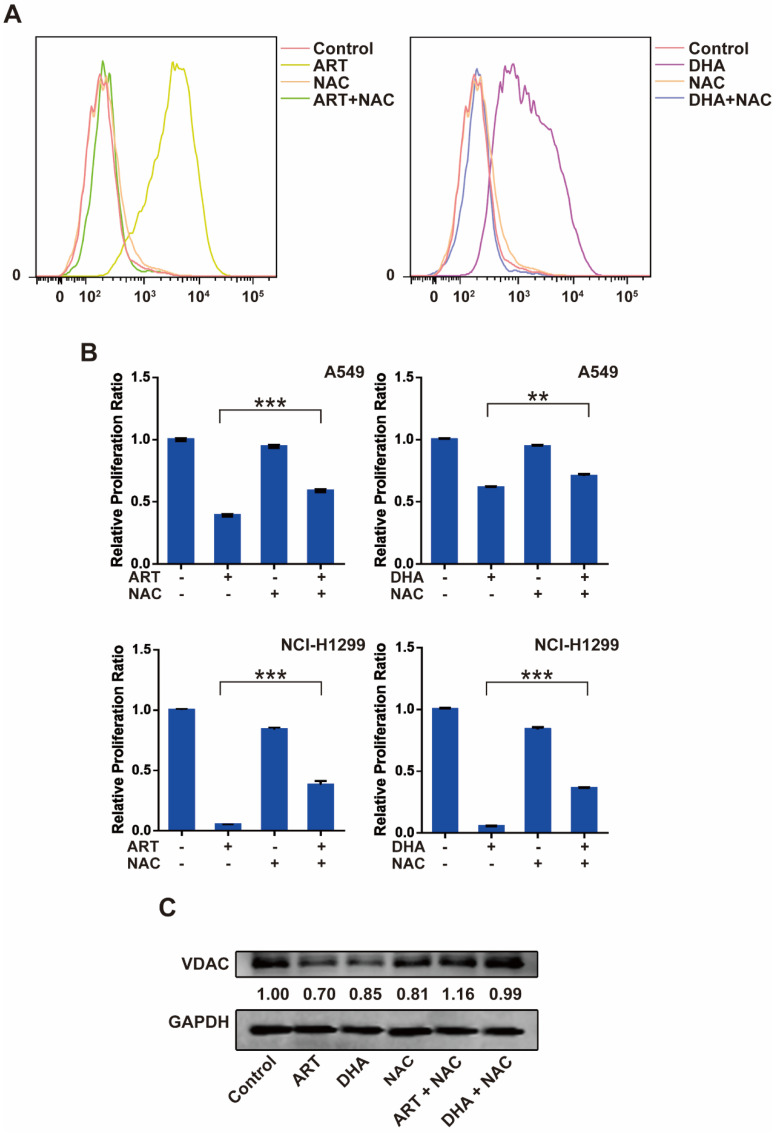

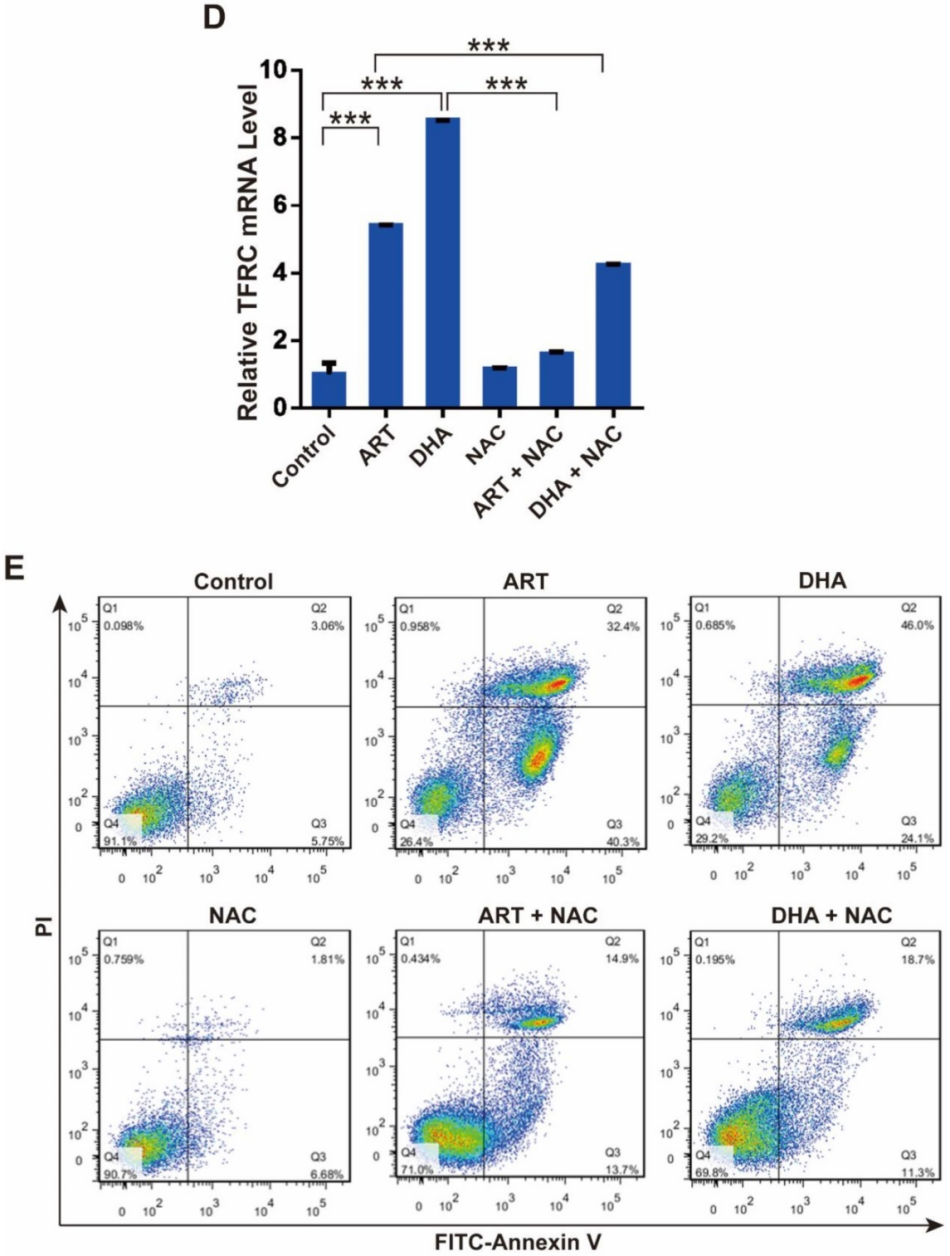
** ROS is a key regulator of ART/DHA-induced apoptosis and ferroptosis. (A)** ART or DHA induced ROS production in A549 cells was observed by flow cytometry, and the ROS production was partly reversed by NAC. A549 cells were treated with ART or DHA (10 μM) and NAC (5 mM) for 72 h. **(B)** The inhibitory effect of ART or DHA on NSCLC cell proliferation was partly reversed by the addition of NAC. A549 or NCI-H1299 cells were treated with ART or DHA (30 μM) and NAC (5 mM) for 48 h.***P < 0.001, **P < 0.01. **(C)** ART or DHA decreased the protein level of VDAC, which was partly reversed by NAC. A549 cells were treated with ART or DHA (10 μM) and NAC (5 mM) for 72 h. ***P < 0.001. **(D)** ART or DHA increased the TFRC mRNA level, which was partly reversed by NAC. A549 cells were treated with ART or DHA (30 μM) and NAC (5 mM) for 48 h. ***P < 0.001. **(E)** ART or DHA induced apoptosis in A549 cells, which was partly reversed by NAC. A549 cells were treated with ART or DHA (30 μM) and NAC (5 mM) for 72 h.

Reactive oxygen species (ROS) plays an important role in cell signal transduction and tissue homeostasis. Excessive ROS triggers the opening of mitochondrial PTP leading to apoptosis and causes lipid peroxidation leading to ferroptosis 19, 20. To investigate whether ROS is a key molecule that mediates artemisinin derivatives' effect on NSCLC cells, we utilized N-Acetyl-L-cysteine (NAC), a ROS scavenger, to block the production of ROS in NSCLC cells. As anticipated, treatment of A549 cells with ART or DHA stimulated the production of ROS, which was reversed partly by NAC (**Figure 4A**). Consistently, the inhibitory effect of artemisinin derivatives on the proliferation A549 and NCI-H1299 cells was partly reversed by the addition of NAC (**Figure 4B**). ART and DHA decreased the protein level of VDAC, which was partly reversed by NAC (**Figure 4C**). The addition of NAC decreased the mRNA level of TFRC induced by ART and DHA in A549 cells (**Figure 4D**). Furthermore, the addition of NAC indeed significantly reversed the apoptosis induced by ART and DHA in A549 cells (**Figure 4E**). These results established that artemisinin derivatives inhibited NSCLC cells through induction of ROS-dependent apoptosis/ferroptosis.

### The gene expression and clinical implication of TFRC and VDAC in lung cancer

**Figure 5:**
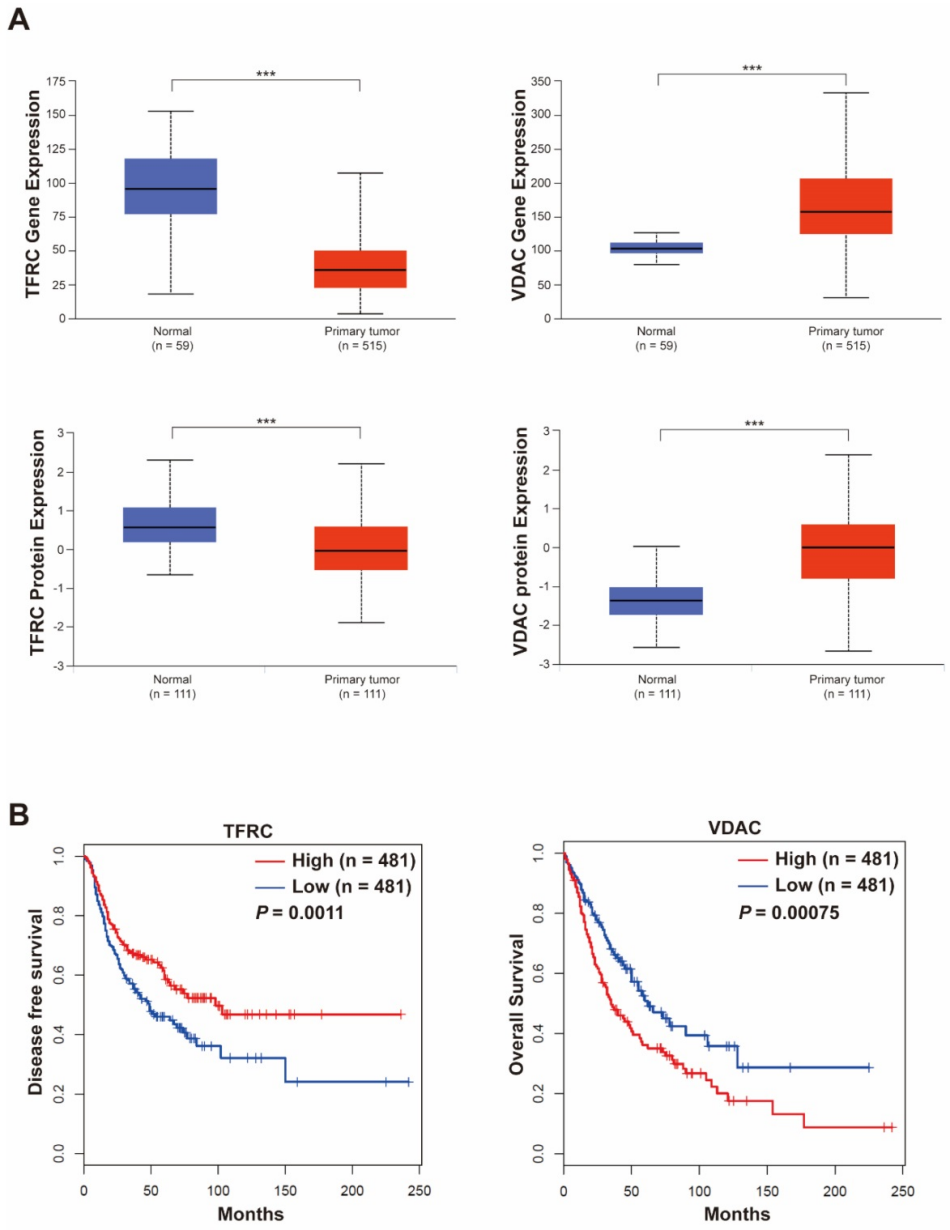
** Gene and protein expression of TFRC and VDAC and their relationship with lung cancer patient survival. (A)** Gene and protein expression of TFRC and VDAC in cancer and normal tissues of lung cancer patients analyzed using the UALCAN database (http://ualcan.path.uab.edu/). ***P < 0.001.** (B)** Relationship between TFRC and VDAC and survival of lung cancer patients analyzed using the GEPIA 2 database (http://gepia2.cancer-pku.cn/#index).

We analyzed the gene and protein expression of TFRC and VDAC in lung cancer patients through the UALCAN database. We found that while TFRC was poorly expressed, VDAC was highly expressed in tumor tissues compared with normal tissues (**Figure 5A**). Then, we explored the relationship between these molecules and the lung cancer patients' survival by GEPIA 2 database. Our results revealed that the expression levels of TFRC and VDAC were positively and negatively correlated with the survival of lung cancer patients, respectively (**Figure 5B**). All these data suggested that TFRC and VDAC were closely associated with the survival of lung cancer patients and can be used as potential therapeutic targets in lung cancer.

## Discussion

This study explored the role of ART/DHA in NSCLC cells and the mechanism involved. Our results indicated that ART/DHA inhibited cell viability in a dose-dependent manner in NSCLC cells. In addition, we found that ART/DHA inhibited NSCLC cells through induction of ROS-dependent apoptosis/ferroptosis. Our study has provided evidence, for the first time, that artemisinin derivatives inhibited NSCLC cells through ROS-mediated dual death pathway.

**Figure 6:**
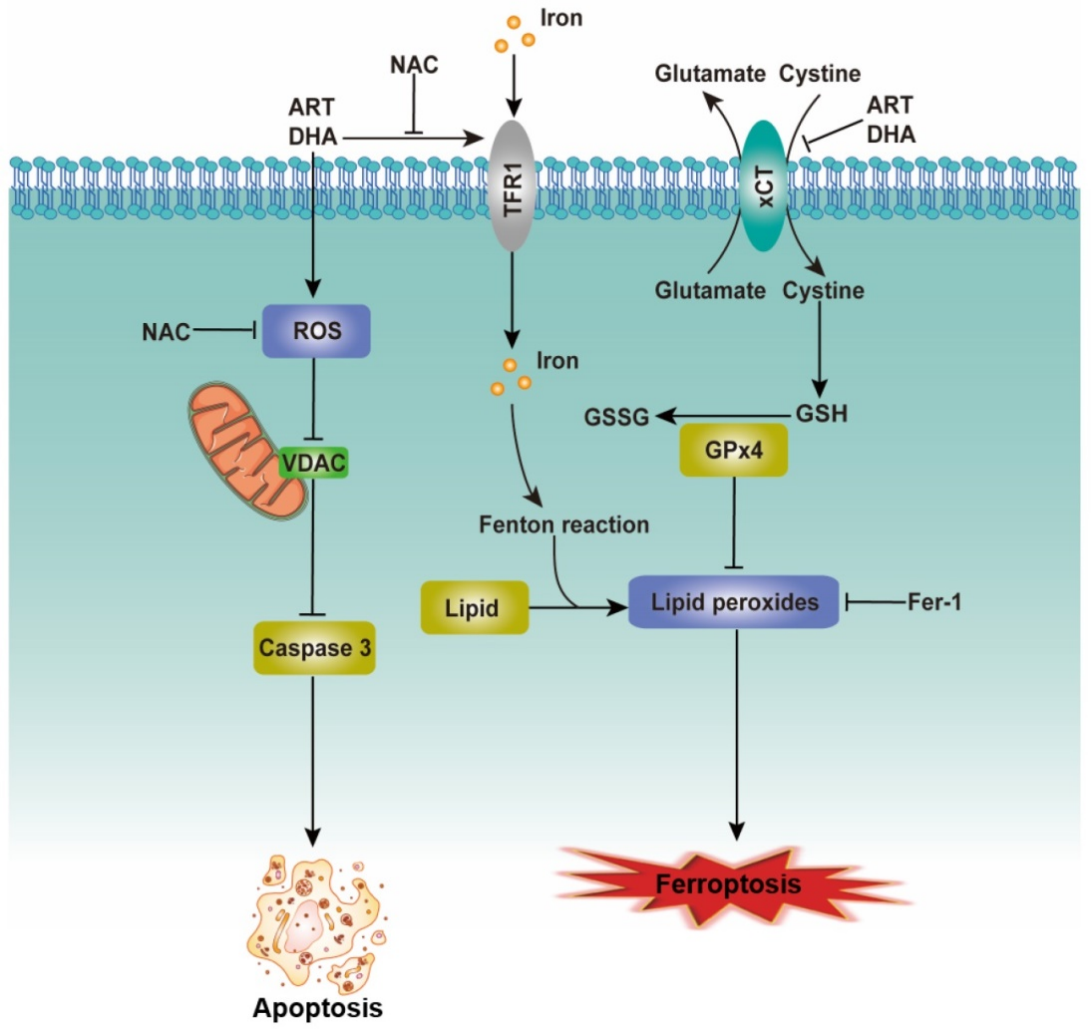
** The proposed mechanism for the inhibitory effect of artemisinin derivatives on NSCLC cells.** See text for detailed description on how artemisinin derivatives induce apoptosis/ferroptosis.

Cell death is important for homeostasis and normal development and is necessary for the maintenance of tissue function and morphology 21. Oxidative stress, the result of excessive accumulation of ROS, can induce the opening of PTP, the release of cytochrome c from the mitochondrion and the activation of a caspase cascade, eventually resulting in apoptosis 20, 22. We provide evidence that ART/DHA increased the ROS level and induced apoptosis in NSCLC cells. VDAC, also known as mitochondrial porin, regulates various cellular processes including apoptosis. In the model in which VDAC is expressed at a low level, the PTPs let solutes enter the mitochondria hence increasing the volume of the mitochondrial matrix, which causes local rupture of the mitochondrial membrane and the release of cytochrome c 23. As anticipated, our study showed that ART/DHA decreased the protein level of VDAC and increased caspase 3 cleavage. It's likely that ART/DHA induces apoptosis through ROS-mediated mitochondrial disruption (**Figure 6, left portion**).

In addition to apoptosis, several other forms of programmed cell death such as ferroptosis have been discovered recently 24. Ferroptosis, characterized by the iron-dependent accumulation of lipid hydroperoxides to lethal levels, is controlled by the xCT/glutathione (GSH)/glutathione peroxidase 4 (GPX4) axis. xCT, a core negative regulator of ferroptosis, is responsible for maintaining redox homeostasis by importing cystine, where it is then reduced to cysteine and used for the synthesis of the major antioxidant GSH 25. The protein level of xCT was downregulated by ART/DHA, which indicated ART/DHA could decrease the antioxidant capacity of cells and induce ferroptosis. TFRC can import extracellular iron into the cell and promotes ferroptosis. Several studies suggested that the expression of TFRC is upregulated in ferroptosis-sensitive cells 25, 26. Interestingly, ART/DHA upregulated the mRNA level of TFRC, which was partly reversed NAC. This study established that artemisinin derivatives inhibit NSCLC cells through induction of ROS-dependent apoptosis/ferroptosis (**Figure 6, right portion**).

Combination therapy approach is commonly used to increase efficacy and overcome drug resistance 27. Artemisinin combination therapy (ACT) as standard treatment of uncomplicated malaria was recommended by WHO 28. Derivatives of artemisinin have been shown to possess selective anti-cancer properties. Therefore, cancer therapy in combination with artemisinin-type drugs is receiving increasing attention recently 29. We used artemisinin derivatives in combination with lapatinib, an inhibitor of receptor tyrosine kinases such as EGFR. Cell viability assay results showed that artemisinin derivatives sensitized NCI-H1299 cells to EGFR inhibitor lapatinib, as demonstrated by combination index < 1 for ART or DHA combined with lapatinib (data not shown). Our study explored a novel mechanism of artemisinin derivatives which provides a new strategy for NSCLC therapy.

In conclusion, through natural compound screening assay, we found that artemisinin derivatives have a significant inhibitory effect on NSCLC cells. Artemisinin derivatives inhibit NSCLC cells through induction of ROS-dependent apoptosis/ferroptosis. Our results provide the experimental rationale for the potential application of artemisinin derivatives as a class of novel therapeutic drugs for NSCLC.
